# Supplementary material for: Collective excitations and low-energy ionization signatures of relativistic particles in silicon detectors
Source: Commun Phys. 2024 Dec 19;7(1):416. doi: 10.1038/s42005-024-01904-2 (PMC11659158; doi:10.1038/s42005-024-01904-2)
Supplement: Supplementary file 1 — Supplementary Material [file 42005_2024_1904_MOESM1_ESM.pdf]

Supplementary Material: Collective excitations and low-energy ionization signatures of relativistic particles in silicon detectors

Rouven Essig,[1], Ryan Plestid,[2], and Aman Singal[1,3]

[1]C. N. Yang Institute for Theoretical Physics, Stony Brook University, Stony Brook, NY 11794, USA
[2]Walter Burke Institute for Theoretical Physics, California Institute of Technology, Pasadena, CA 91125, USA
[3]Institute for Advanced Computational Sciences, Stony Brook University, Stony Brook, NY 11794, USA

Supplementary Note 1. Additional figures for momentum-weighted energy loss function

In this appendix, we provide additional figures, which we feel may be helpful in interpreting our results. In Fig. S1, we show a slice of the momentum-transfer-weighted energy loss function at a fixed energy transfer of $\omega = 45$ eV. This is the integrand in Eqs. (1) to (3) for different interactions, ranging from a massless mediator (k^{-1}), to a neutrino dipole moment (k^{1}), to a contact operator (k^{3}). In Fig. S2, we show the same momentum weightings, but at the level of the integral as a function of ω. These curves dominate the relativistic energy loss formulae, Eqs. (1) to (3), although they receive additional corrections from the transverse modes for relativistic kinematics.

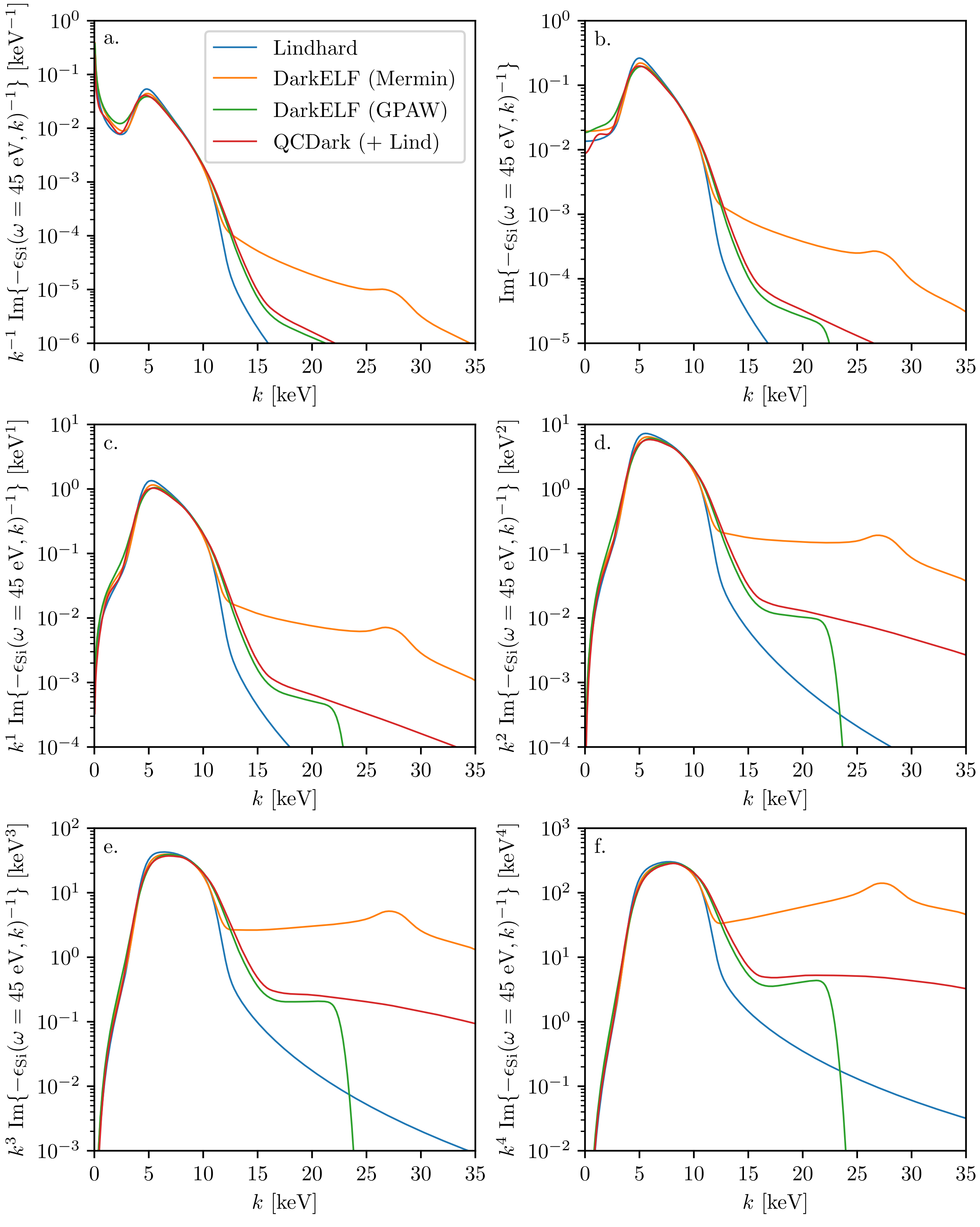

Figure S1: SupplThe wavenumber dependence of the silicon dielectric function calculated with various approximations and numerical techniques. The electron loss function, Im$\{-\epsilon(\omega, k)^{-1}\}$ has been smoothed by averaging over the energy axis in a 2 eV bin centered at $\omega = 45$ eV, and underwent Gaussian smoothing on the k axis with $\sigma_k = 0.5$ keV. `QCDark` shows the effect of *all-electron* inclusions at high $k \gtrsim 23$ keV, while the Mermin function in `DarkELF` overestimates the imaginary part of $\epsilon(\omega, k)$ for $k \gtrsim 11$ keV.

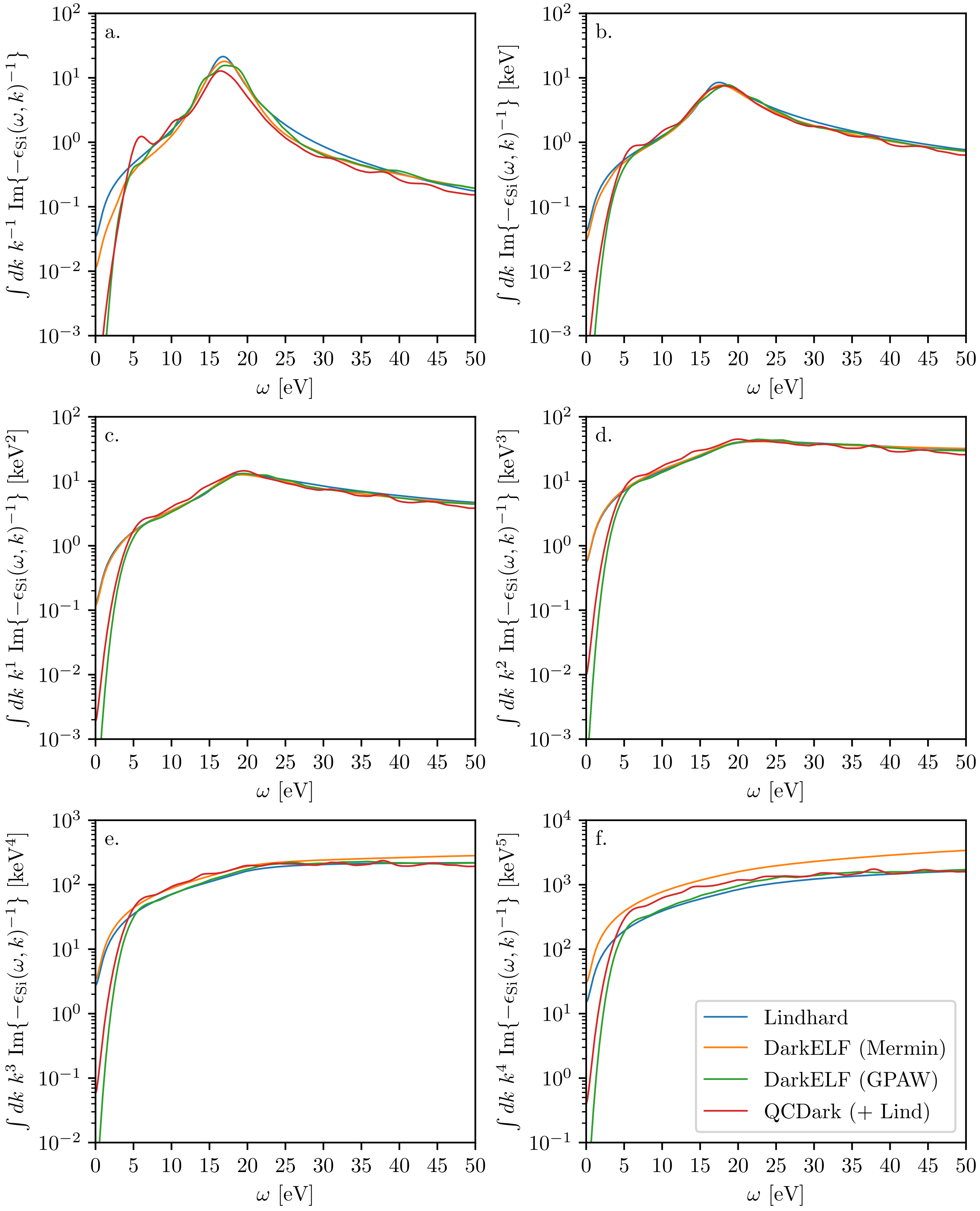

Figure S2: The frequency dependence of $\int dk\, k^n\, \mathrm{Im}\left\{-1/\epsilon(\omega,k)\right\}$ for the silicon dielectric function calculated using various codes. The results have undergone a Gaussian smoothing with $\sigma_\omega = 0.5$eV.
