# Supplementary material for: Collective excitations and low-energy ionization signatures of relativistic particles in silicon detectors
Source: Commun Phys. 2024 Dec 19;7(1):416. doi: 10.1038/s42005-024-01904-2 (PMC11659158; doi:10.1038/s42005-024-01904-2)
Supplement: Supplementary file 2 — Description of Additional Supplementary Files [file 42005_2024_1904_MOESM2_ESM.pdf]

**Description of Additional Supplementary Files**

File name- Supplementary Data 1

File description- All data shown in all figures in the main manuscript file.
